# JUNB and JUND in Urological Cancers: A Literature Review

**DOI:** 10.3390/cimb47090741

**Published:** 2025-09-10

**Authors:** Georgios Kalampounias, Theodosia Androutsopoulou, Panagiotis Katsoris

**Affiliations:** 1 Laboratory of Cell Biology, Division of Genetics, Cell and Developmental Biology, Department of Biology, School of Natural Sciences, University of Patras, 26504 Patras, Greece; gkalampounias@ac.upatras.gr (G.K.); tandroutsopoulou@ac.upatras.gr (T.A.); 2 Institute for Bioinnovation, Biomedical Sciences Research Centre “Alexander Fleming”, 16672 Athens, Greece

**Keywords:** JUN, c-JUN, JUNB, JUND, JNK, MYC, FOS, prostate cancer, bladder cancer, renal cell carcinoma

## Abstract

JUNB and JUND are two transcriptional factors (TFs) of increased interest in cancer, regulating the expression of genes associated with survival, proliferation, differentiation, migration, invasion, angiogenesis, adhesion, apoptosis, and cell cycle regulation. Together with c-JUN, they constitute the JUN family of TFs, acting as downstream effectors of the MAPKs, with established roles in carcinogenesis, disease progression, metastasis, and therapy resistance. Their phosphorylation leads to the formation of dimeric complexes with other TFs (from the JUN, FOS, or ATF families), thereby assembling the AP-1 complex, which exerts multifaceted influences on both normal and cancerous cells. JUNB and JUND are credited with both tumor-suppressing and oncogenic roles, since the outcome of their activation relies on the specific cancer type, disease stage, intracellular localization, and the expression of interacting cofactors. This narrative review explores the current understanding of JUNB and JUND roles within urological cancers (prostate, bladder, renal, and testicular cancer) as these malignancies, while distinct, share common genetic and/or environmental risk factors and varying degrees of androgen receptor (AR) dependency. The study discusses commonalities and differences in the expression patterns, mechanisms, and clinical implications of JUNB and JUND across urological cancers, thus highlighting their potential as prevention, diagnosis, prognosis, and treatment targets.

## 1. Introduction

The genitourinary system consists of several organs grouped together not only due to their proximity and interconnection in the mammal body, but also due to the organ’s common embryological origins and subsequent cellular, molecular, and biochemical commonalities [1,2]. Both parts of the system, namely the urinary and the reproductive organs, are composed of cells of high turnover, highly responsive to hormones and extracellular stimulants, and exposed to carcinogens [3,4,5]. These parameters, intermingled with genetic and environmental risk factors unique for every individual, render the system susceptible to the emergence of malignancies which are the leading cause of death for thousands of patients every year [5]. Although the organs have a common ancestry from a developmental biology perspective, excessive differentiation and key differences between the two genders divide the study of genitourinary cancers into the separate fields of gynecological and urological oncologists. The urological malignancies grouped account for more than 13.2% of total cancer cases [6], thus underscoring the significance of their study and improvement of our treatment options. This review article focuses on the four most common types of urological cancer, namely prostate, bladder, renal, and testicular cancer. Prostate cancer (PCa) is the second most common type of cancer diagnosed among men and the fifth leading cause of cancer death globally. PCa is responsible for 1,466,680 new cases and 396,792 deaths in 2022 and accounted for the 7.3% of total cancer cases and 4.1% of all cancer-related deaths in that year [6]. Bladder cancer (BCa) is also a very common type of cancer (ranking ninth globally), affecting both genders and being responsible for 613,791 new cancer cases and 220,349 deaths in 2022 [6,7]. BCa accounted for 3.1% of all cancer cases in 2022 and 2.3% of all cancer-related deaths [6]. Cancer of the kidneys (or renal cancer) affected 434,419 patients in 2022 and was responsible for 155,702 deaths in the same year, ranking fourteenth internationally [6]. Finally, 72,031 new diagnoses of testicular cancer were made in 2022, and 9056 men lost their lives to it, being the 27 th most common type of cancer globally [6]. Although urological cancers are leading causes of death for hundreds of thousands of individuals, and considerable amount of research has been conducted [8,9,10,11,12], current understanding is still limited by the multifaceted nature of cancer.

Each of the urological cancer types or even subtypes follows distinctive therapeutic protocols [7,13,14,15]; however, several underlying disease emergence/progression mechanisms are common due to the high similarity at the genomic, epigenomic, transcriptomic, proteomic, and metabolomic levels. Major oncogenes such as mutated *RAF* and *RAS* alleles, *MYC*, and the AP-1 complex subunits *FOS*, *ATF*, and *JUN* are reported to be active in the majority of the cases and regulate processes like angiogenesis, metastasis, and chemoresistance [16,17,18,19,20,21,22,23,24,25], which render the disease aggressive and ultimately incurable [12,26,27,28,29]. Since the underlying mechanisms often vary and each cancer case has a distinctive proteomic profile, it is important to integrate existing knowledge on molecules of high diagnostic, prognostic, and even therapeutic value. Given the importance of the JUN family of transcription factors (TFs) in the development of the disease [16,17,18,19,20,21,22,23,24], this review focuses on the less studied JUNB and JUND and comprehensively presents and synthesizes current knowledge. Compared to c-JUN, far less data exists on JUNB and JUND; however, their various implications in multiple disease aspects could provide us with novel tools and strategies in the fight against urological cancers and all malignancies in general.

## 2. Structural and Regulatory Characteristics of the JUN Family of Transcription Factors

### 2.1. Commonalities and Differences Between the Three JUN Family Proteins

The JUN family of TFs consists of three members: c-JUN, JUNB, and JUND, and all of them can form homodimeric or heterodimeric (with other JUN, FOS, or ATF proteins) structures that act as transcription regulators. The dimer, namely AP-1, depending on the two subunits and the expression of TFs, can act as a tumor suppressor or promoter and has been found to regulate the expression of several downstream genes [30,31,32,33,34]. JUN TFs are activated via phosphorylation at specific sites, which stabilizes them and prevents degradation by ubiquitination, while phosphorylation at different sites results in a higher turnover rate [35,36,37,38,39,40].

**Figure 1:**
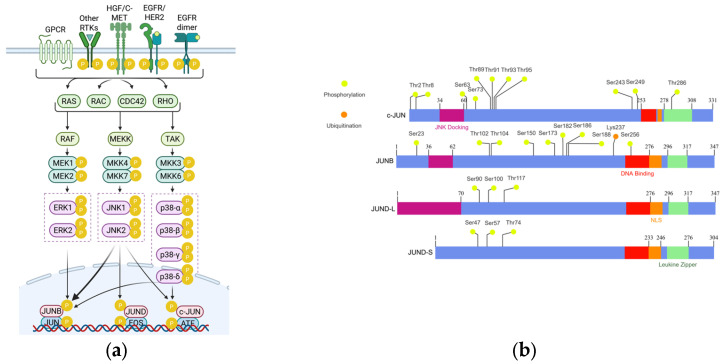
Activation pathways, structure, and post-translational modification sites of c-JUN, JUNB, and JUND. (**a**) The phosphorylation of c-JUN, JUNB, and JUND is mediated mostly by MAPKs. The figure illustrates how different MAPKs specialize to each JUN-family protein as well as upstream molecules. C-JUN, JUNB, and JUND can form dimers with other JUN proteins, c-FOS, or ATF transcription factors and thus form AP-1. Different monomer combinations and post-translational modifications regulate whether AP-1 will be a promoter or a suppressor of gene expression. Created in BioRender. Kalampounias, G. (2025) https://BioRender.com/35c80gh. (**b**) The structure of c-JUN, JUNB, and JUND exhibits several similarities and distinctive attributes. Most studied phosphorylation and ubiquitination sites are displayed as well as the regions responsible for JNK docking, nuclear translocation, DNA binding, and dimerization. The figure was created with IBS 2.0.

Regarding their activation, JUN proteins are mostly phosphorylated by the Jun N-terminal kinases (JNKs), which are a group of Mitogen-activated protein kinases (MAPKs) that can both activate or suppress the activity of JUN family members [41,42]. Moreover, the other MAPK groups, Extracellular signal kinases (ERKs) and the p38 MAPKs, have also been found to phosphorylate JUN TFs, exerting both activating and suppressing effects on them [35,40,43,44,45,46,47,48,49]; however, to a lower extent due to structural limitations (Figure 1a). Besides MAPKs, other kinases have also been found to regulate the phosphorylation of JUN, including Protein kinase C (PKC) [49,50], Casein kinase 2 (CK2) [49], p21-activated protein kinase (PAK2) [51], as well as Vaccinia-related kinase 1 (VRK1) [52], and C-terminal Src kinase (CSK) [39]. c-JUN is the most studied member of the three, and the vast majority of studies credit it with oncogenic activities. c-JUN phosphorylation has been reported in various sites; however, the most well-characterized phosphorylation sites are Ser^63^ and Ser^73^, near the NH_2_ terminus [53]. This type of phosphorylation has been correlated to JNK activity and mostly results in transcriptional activation and c-JUN stability [39,53]. Other sites have been associated with pro-apoptotic activity (such as Thr^91^, Thr^93^, and Thr^95^) [54], while others are correlated to c-JUN’s repression (such as Ser^243^) [50,55]. The major well-known phosphorylation sites of c-JUN and the structure of the protein can be found in Figure 1b, which was prepared using IBS 2.0 [56]. JUNB and JUND are not equally well characterized and are mostly credited with effects opposing those of c-JUN [36,40,57,58,59]. However, recent studies have come up with findings that do not always support an opposing role but also indicate synergy [36,60,61,62]. All three members, depending on the phosphorylation sites, the presence of other AP-1-forming monomers, and the interaction with other TFs, can either upregulate or downregulate the transcription of specific genes [32,35,63,64]. This study focuses on JUNB and JUND and summarizes current knowledge on both factors in the context of urological cancers.

### 2.2. Structure and Post-Translational Modifications of JUNB

The gene encoding the JUNB protein (*JUNB*) is located on human chromosome 19p13.13 and has no introns. The protein consists of 347 amino acids and has a molecular weight of about 35.9 kDa. Structurally, it contains a JNK docking site, a nuclear localization signal, a basic domain for DNA binding, and a leucine zipper domain for dimerization (Figure 1b) [35,65]. At its C terminal there is a DNA-binding domain, and at its N terminal there is a transcription activation domain [35]. A major parameter in JUNB-mediated gene transcription is its upstream regulation. Several epigenetic, transcriptional, and post-transcriptional modifications have been reported; however, these will be discussed in the context of urological cancers in the next sections [35,37,38,66]. An important part of JUNB’s regulation that has not been exclusively studied in the context of urological cancers is that of post-translational modifications. JUNB has been found to undergo several post-translational modifications that regulate its stability, turnover rate, DNA-binding capacity, and its ability to promote gene transcription [35]. It can be phosphorylated by JNKs (Thr^102^ and Thr^104^) [67], by ERK1/2 (Ser^256^) [46], by p38 (no reported site) [44,45], by CDK complexes (Ser^23^, Thr^150^, and Ser^186^) [40,43], and by ABL1 (Tyr^173^, Tyr^182^, and Tyr^188^) [68] (Figure 1a). Regarding JNK-induced activation, it has not yet been given a definite answer, since some studies support that JNK can indeed phosphorylate JUNB [36,40,48,66]; however, other groups reported that JNKs cannot phosphorylate JUNB since the JNK-activating domain has not been conserved in mammals [69]. A more recent study by Kanamori et al. in 2018 reported that IL1B induced the transcription of hepcidin via the activation of JUNB and not c-JUN [48]. Moreover, activation of JUNB by JNKs has been reported in zebrafish [47]. Overall, phosphorylation by JNKs and ERK1/2 usually activates transcriptional activation [46,47,48,67], while phosphorylation by CDKs promotes its inactivation by degradation [40,43]. Destabilizing are also the effects of ABL1 phosphorylation [68]. The destabilization of JUNB and its subsequent degradation is facilitated upon ubiquitination (Figure 1b), while the ubiquitination is also promoted by another post-transcriptional modification, NEDDylation [37]. On the contrary, SUMOylation of JUNB has been found to promote its transcriptional activity, as reported by Garaude et al. [38] in 2018.

### 2.3. Structure and Post-Translational Modifications of JUND

Regarding JUND, the gene encoding (*JUND*) is intronless [70] and is located on human chromosome 19p13.11, and the resulting protein has 347 amino acids and an approximate molecular weight of 35.2 kDa (Figure 1b). Near its N-terminus, JUND has a JNK-phosphorylating motif (Ser^90^/Ser^100^), a domain for DNA binding, an NLS domain, and a leucine zipper domain that allows dimerization. According to Kallunki et al. [69] in 1996, the gene lacks a docking site for the JNKs, which translates into reduced JNK-mediated phosphorylation (compared to c-JUN) [69,71]; however, phosphorylation by JNKs has been reported in several studies [69,71,72,73,74,75,76] (Figure 1a). The protein appears in two isoforms, JUND-L and JUND-S, depending on the in-frame translational initiation site [77]. The shorter isoform (JUND-S) lacks 43 amino acids from its N-terminus, rendering the interactions between JUND-S and menin (MEN1) unfeasible [72,77]. Regarding JUND’s phosphorylation sites, Ser^90^, Ser^100^, and Thr^117^ have been recognized as MAPK targets, primarily through JNK activation [78], while ERK1/2 have also been identified as JUND activators [73] (Figure 1b). The tumor suppressor MEN1 modulates JUND activation by all MAPKs [78]. JUND has been shown to act independently from p38 MAPKs (while c-JUN is a target of p38), as reported by Humar et al. in 2007 [44]. Besides phosphorylation, SUMOylation has also been reported as a post-translational modification affecting JUND’s activity [79]. SUMOylation and inhibition of MAPK-dependent JUND phosphorylation are the two pathways suggested to explain JUND repression by MEN1 [79]. SUMOylation is catalyzed following the formation of the MEN1–JUND complex, and according to Hernandez et al., it is a crucial step in the mSin3A–histone deacetylase (HDAC) recruitment, which facilitates JUND suppression [79]. Finally, JUND has been found to undergo reduced ubiquitination and has a fourfold higher half-life compared to c-JUN [80].

## 3. Prostate Cancer

### 3.1. JUNB in Prostate Cancer

In the context of prostate cancer (PCa), JUNB has been reported to exhibit mostly tumor-suppressing functions. Over the years, several mechanisms involving JUNB have been reported, mostly highlighting its activity as a c-JUN antagonist. JUNB antagonizes c-JUN and forms suppressive AP-1 complexes with other TFs, thus exhibiting mostly antiproliferative and antimetastatic activity (Table 1). However, there are several studies that credit JUNB with a pro-tumorigenic role, having similar gene targets with c-JUN and being a promoter of proliferation, epithelial-to-mesenchymal transition (EMT), and metastasis. All these findings combined underscore the significance of co-expressed factors and transcription regulators, which can differ among different PCa subtypes, disease stages, or even subclones of the same tumor.

**Figure 2:**
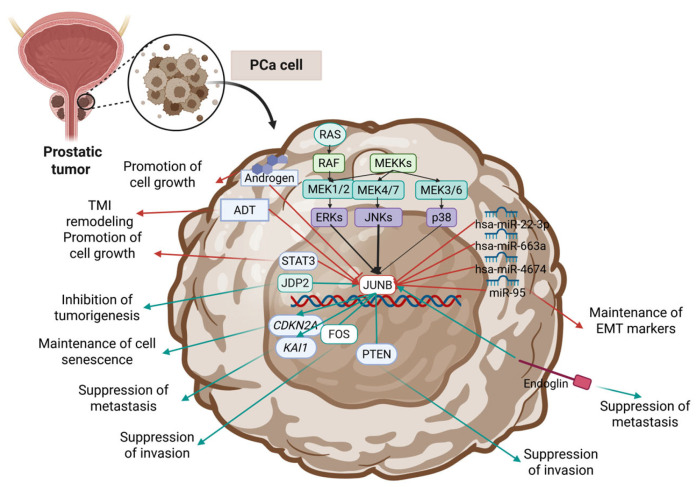
Key mechanisms of JUNB’s action and regulation reported in PCa studies. Blue arrows correspond to tumor-suppressing mechanisms, and red arrows correspond to oncogenic mechanisms. Created in BioRender. Kalampounias, G. (2025) https://BioRender.com/hz3v7gw.

Strong data crediting JUNB with a tumor-suppressor role in PCa was reported in 2008 by Konishi et al. [81], while indications about this phenomenon were also available some years prior. In this study, JUNB was credited with the maintenance of cell senescence in the transient amplifying cells (TACs) of the prostate by promoting the expression of cell cycle regulators such as p16 [81] (Figure 2). The authors reported that the expression of JUNB was negatively correlated to the cells’ invasiveness, while JUNB silencing led to cell clones with prominent invasiveness and metastatic potential [81]. The connection between JUNB’s loss/downregulation and invasiveness was also confirmed by later studies [33,82,83]. A 2015 study by Thomsen et al. reported that in low-grade PCa, JUNB is highly expressed compared to normal tissues, while in high-grade, metastatic PCa, JUNB’s expression is significantly downregulated [82]. The loss of JUNB and Phosphatase and tensin homolog (PTEN) in mouse models led to highly invasive PCa, and decreased levels of cyclin-dependent kinase inhibitor 2A (CDKN2A or p16^INK4a^) and Cyclin-dependent kinase inhibitor 1A (CDKN1A or p21^Waf1/Cip1^) were documented [82] (Figure 2). JUNB’s role in senescence has also been mentioned in other cancer types, in which similar mechanisms have been described [58,84,85,86]. A study by Riedel et al. confirmed that in advanced PCa stages, the expression of JUNB and Fos proto-oncogene, AP-1 transcription factor subunit (FOS), are reduced [33]. The authors investigated how the loss of JUNB/FOS can affect the proliferation of PCa cells [33]. Loss of JUNB or FOS in PTEN cells results in proliferation promotion, while the loss of FOS alone leads to an upregulation of JUNB levels [33] (Figure 2). The inactivation of JUNB was also shown to promote pro-invasive activity, thus crediting JUNB with a tumor suppression role in early PCa stages, which is gradually lost in later stages [33]. Bioinformatics studies on transcriptomic data have repeatedly reported the downregulation of JUNB in advanced PCa stages and in metastatic disease [87,88]. A 2011 study reports that JUNB participates in the anti-metastatic process by promoting the expression of the metastasis suppressor gene *KAI1* [89] (Figure 2). The positive correlation between *JUNB* and *KAI1* expression had been reported by previous studies [90]; nonetheless, in this study JUNB was found to cooperate with ATF3 [89], a transcription factor that can act both as an activator or a suppressor of metastasis depending on the cellular environment and the cell type [91,92,93]. Another 2011 study by Lakshman et al. investigated the mechanisms underlying Endoglin’s anti-metastatic potential and showed that it promotes the activation of Smad1 and Smad1-responsive genes such as JUNB, signal transducer and activator of transcription 1 (STAT1), and SRY-box transcription factor 4 (SOX4) [94] (Figure 2). Finally, a very interesting finding regarding JUNB’s presence and prognostic value was reported in 2024 by Roumeliotou et al., in which JUNB was detected in circulating tumor cells (CTCs) from metastatic prostate cancer cases, in a percentage as high as 28%, from a sample of 48 patients [95]. The authors also reported that JUNB phenotypes (CK+/CXCR4+/JUNB-) are linked to a poor prognosis since they were correlated to poorer progression-free survival [95].

**Table 1:** Roles of JUNB in prostate cancer.

Cancer	Model	Role	Mechanism	Effect	Ref.
PCa	Patient-derived tissues and human PCa cell lines	Tumor suppressor	Upregulation of p16 ^INK4a^	Senescence maintenance	[81]
PCa	Patient-derived PCa cells and mice	Tumor suppressor	Cooperation with PTEN	Invasion suppression	[82]
PCa	Human PCa cell lines and mice	Tumor suppressor	Cooperation with FOS	Invasion suppression	[33]
PCa	Bioinformatics study	Tumor suppressor	Low JUNB levels in metastatic PCa are correlated to a poor prognosis	Metastasis biomarker	[87,88]
PCa	Human PCa cell lines (DU-145, PC-3, PZ-HPV-7, LNCaP)	Tumor suppressor	Positive regulation on the *KAI1* gene	Metastasis suppression	[89,90]
PCa	Androgen-independent human PCa cell line (PC-3)	Tumor suppressor	Endoglin activates Smad1-responsive genes (JUNB, STAT1, and SOX4)	Metastasis suppression	[94]
PCa	PCa-derived circulating tumor cells (CTCs)	Tumor suppressor	JUNB is present in 28% of PCa CTCs. CK+/CXCR4+/JUNB- phenotypes are correlated to poor prognosis	Metastasis biomarker	[95]
PCa	Human PCa cell lines (DU-145, PC-3, LNCaP) and mice xenografts	Tumor suppressor	The expression of JUNB increases as a result of JDP2	Tumorigenesis inhibition	[96]
PCa	Patient-derived PCa tissues	Tumor suppressor	JUNB, KMT2A, and XPO6 are downregulated by hsa-miR-22-3p, hsa-miR-663a, and hsa-miR-4674 in mPCa	Metastasis suppression	[97]
PCa	Patient-derived PCa tissues and human cell lines	Tumor suppressor	miR-95 of exosomal origin targets JUNB	EMT Maintenance	[98]
PCa	Androgen-independent human PCa cell line (DU-145)	Oncogene	Similarly to c-JUN, it contributes to the expression of FGF1, PTPN5, ADAM19, SERPINE1, CXCR7, MMP9, PLAU, and PTHLH	Migration promotion	[99]
PCa	Patient-derived PCa tissues, human PCa cell lines (DU-145, PC-3, LNCaP) and mice	Oncogene	CDK5 activates STAT3, which in turn upregulates *JUNB*, *FOS*, *MYC*, and *Survivin* (*BIRC5*)	Cell growth	[100]
PCa	Patient-derived PCa tissues	Oncogene	ADT induces the activation of ZFP36, JUNB, and SOCS3	TMI remodeling	[101]
PCa	Androgen-independent human PCa cell lines (DU-145, PC-3M)	Oncogene	*GADD45B*, *CTGF*, and *JUNB* are induced by the Smad3-EPSM	Metastasis promotion	[102]
CRPC	Patient-derived PCa tissues	Oncogene	TERC, MYBL3, HRAS, PI3KCA, LAMC2, RAF1, MYC, GARP, SAS, FGFR1, PGY1, MYCL1, MYB, FGR, and JUNB are found amplified in CRPC	Disease progression	[103]
CRPC	Patient-derived PCa tissues	Oncogene	A TFCG enriched in mCRPC contains JUN, JUNB, JUND, FOS, FOSB, and FOSL1	Disease progression	[104]
CRPC	Rat ventral prostate epithelial cells	Oncogene	Androgen suppresses JUNB expression, while the loss of hormone dependency fuels its activation	Proliferation promotion	[105]

Abbreviations: PCa = prostate cancer; EMT = epithelial-to-mesenchymal transition; CRPC = castrate-resistant prostate cancer; CTCs = circulating tumor cells; ADT = androgen-deprivation therapy; TMI = tumor immune microenvironment; TFCG = transcription factor coordinated group.

Regarding JUNB’s regulation, a 2021 study by Raspin et al. identified a rare variant of Enhancer of zeste homolog 2 (EZH2), rs78589034, as a significant risk factor [106]. Disruptions in EZH2 function were shown to affect the expression of *JUNB* as well as Dual specificity phosphatase 1 (*DUSP1*), *FOS*, and Early growth response 1 (*EGR1*), and the rs78589034 variant was correlated to increased PCa risk [106]. An older study by Selvaraj et al. in 2015 investigated the regulation of JUN family members phosphorylation in PCa and concluded that Extracellular signal-Regulated Kinases (ERK) signaling acts as a dynamic switch [99]. The authors credited JUNB with a similar role to that of c-JUN, being able to promote migration upon stimulation, while JUND was credited with antagonizing functions, acting as a suppressor of migration upon activation by Ras-Raf-MEK-ERK signaling [99]. Additionally, a study on androgen-dependent LNCaP cells correlated activated signal transducer and activator of transcription 3 (p-Ser^727^-STAT3) to the expression of JUNB, thus identifying JUNB as a downstream target of STAT3-mediated transcriptional activation [100] (Figure 2). The antagonizing effects between the JUN family members had already been documented in PCa cell lines in a 2004 study by Heinrich et al. [96]. In that study, the Jun dimerization protein (JDP2) was found to act as a suppressor of cell transformation, and as a mechanism, the promotion of JUNB, JUND, and FOS-related antigen 2 (FOSL2; also commonly known as FRA2) expression and the simultaneous downregulation of c-JUN was proposed [96] (Figure 2). The regulation of JUNB by oncogenic micro-RNAs has also been reported as a pro-tumorigenic mechanism. In 2016, JUNB (as well as Lysine methyltransferase 2A [KMT2A] and Exportin 6 [XPO6]) was reported as a target of the miRs hsa-miR-22-3p, hsa-miR-663a, and hsa-miR-4674 [97] (Figure 2). These microRNAs were found to be upregulated in the plasma of mPCa patients compared to plasma levels of patients with a non-metastatic PCa [97]. JUNB has also been reported as a downstream target of miR-95 in PCa [98]. Guan et al. in 2020 reported that exosomic miR-95, derived from tumor-associated macrophages (TAMs), can act as an oncogene by suppressing targeting JUNB of PCa cells, thus promoting cell proliferation, invasion, and the expression of epithelial-to-mesenchymal transition (EMT) markers [98] (Figure 2).

However, JUNB has also been reported as a pro-tumorigenic factor by some studies. Although these types of findings may seem contradictory, in other cancer types (even of urological origin), similar roles for JUNB have been described, thus attributing to it both oncogenic and tumor-suppressing properties. A 2025 study by Wang et al. used single-cell-sequencing data from six PCa cases with Mendelian randomization (MR) analysis and identified a set of genes, including JUNB, of high significance in PCa progression [107]. A 2020 study credited JUNB (also Zinc finger protein 36 [ZFP36] and Suppressor of cytokine signaling 3 [SOCS3]) with a pivotal role in the androgen-deprivation-therapy-induced (ADT-induced) tumor immune microenvironment (TIM) remodeling and also correlated its expression to PSA recurrence-free survival (PSA-RFS) [101]. Coregulations of SOCS3, STAT3, and JUNB have been reported in different contexts as well, thus underscoring the importance of the mechanism in cancer progression [108,109,110,111,112,113,114]. JUNB was also credited with pro-metastatic roles in PCa in a 2018 study by Park et al. [102]. The authors report that mutated SMAD family member 3 (SMAD3-EPSM) increases PCa invasiveness and promotes the expression of EMT drivers [102]. Among those genes, JUNB is reported as a promoter of cell motility and invasion, thus crediting it with a pro-metastatic activity [102] (Figure 2). The development of androgen-independent PCa (also known as castration-resistant prostate cancer, CRPC) has also been correlated to altered JUNB expression [103,104]. A 2003 study by Edwards et al. reports that *JUNB* is among a set of 15 genes (*TERC*, *MYBL3*, *HRAS*, *PI3KCA*, *JUNB*, *LAMC2*, *RAF1*, *MYC*, *GARP*, *SAS*, *FGFR1*, *PGY1*, *MYCL1*, *MYB*, *and FGR*) found upregulated in PCa cases during hormone escape [103]. This correlation was also confirmed by a later study, in which *JUNB* is described as a gene undergoing androgen-dependent regulation. The loss of this type of regulation drives the development of androgen-independent growth [105]. *JUNB* and *JUND* have been identified as hub genes in the process, participating in transcription factor coordinated groups (TFCGs) with a high impact in the transition from hormone-dependent to CRPC [104].

### 3.2. JUND in Prostate Cancer

Several studies have investigated the role of JUND in PCa; nonetheless, no definite answer has been given on whether its role is mostly oncogenic or tumor suppressing, since there are findings supporting both directions. Several studies (of prostate and non-prostate origin) converge that JUND, similarly to JUNB, displays both tumor promoter and tumor suppressor activities regarding several factors, including the cancer type, cellular context, and cooperation with other TFs [32,115,116,117,118,119,120,121,122,123,124] (Table 2). Although JUND and JUNB are often credited with similar tumor promoter/suppressor roles regarding the cellular context, the vast majority of published data (including those in cancer of non-prostatic origin) credit JUND/JUNB with opposing roles to c-JUN [24,99,118,125,126,127]. Although the gene targets of c-JUN, JUNB, and JUND mostly coincide as downstream targets of the AP-1 complex and are similarly regulated by all JUN family members, there are exceptions to this rule [99]. There are groups of genes the regulation of which has been found to be regulated oppositely by c-JUN and JUND [99]. JUND has been mostly reported to be an anti-metastatic factor; however, under certain circumstances, such as constitutive Ras activation and ERK1/2-mediated signaling, JUND can act as a pro-metastatic factor [99]. This role for JUND as a promoter of migration and invasion has also been reported in other cancer types [128,129,130,131,132], thus highlighting its targeting as a potential antimetastatic approach.

**Figure 3:**
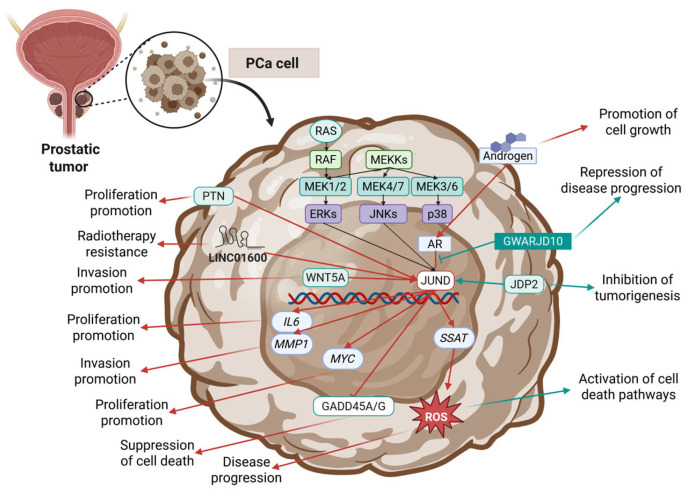
Key mechanisms of JUND’s action and regulation reported in PCa studies. Blue arrows correspond to tumor-suppressing mechanisms, and red arrows correspond to oncogenic mechanisms. Created in BioRender. Kalampounias, G. (2025) https://BioRender.com/bd8c1oo.

As mentioned earlier, JDP2 was also found to be a regulator of JUND’s expression, promoting its activation (together with JUNB) to form inhibitory AP-1 complexes as opposed to tumor-promoting c-JUN-containing AP-1 [96] (Figure 3). Therefore, JUND is theorized as an inhibitor of cell transformation, suppressing the development of PCa by directly antagonizing c-JUN [96]. In a later study, JUND was reported to be implicated in G_1_ cell cycle arrest in androgen-sensitive LNCaP cells, following stimulation by androgen [133]. Androgen was reported to change AP-1 activity following 96 h after exposure, leading to increases in JUND-AP-1 and concomitant decreases in FRA2-AP-1 [133]. The authors associated this expression shift with androgen-induced differentiation activity, probably mediated by JUND, which was theorized to inhibit proliferation in favor of a differentiated prostate phenotype [133]. In a 2008 study by Mahraein-Ghomi et al., JUND was found to be a regulator of reactive oxygen species (ROS) signaling in androgen-dependent LNCaP cells [134] (Figure 3). Non-functional or absent JUND were correlated with lower ROS levels following stimulation by androgen, while even without androgen stimulation, cells overexpressing JUND had higher baseline ROS levels [134]. The same group of authors, in their previous study, had also associated androgen stimulation with increases in JUND activity and ROS levels [133]; however, this 2008 study indicated that JUND acts as a hub protein linking the processes [134]. Given the fact that JUND is correlated to proliferation inhibition and cytostatic effects, this increase in ROS levels could be a part of the regulatory mechanism causing cell cycle arrest while promoting stress responses and differentiation [134]. However, excessive ROS production can be a PCa driver by fueling both carcinogenesis and disease progression (Figure 3). A later study by the same group showed that upon activation of the AR by androgen, JUND forms a complex with it, and it can induce the expression of Spermidine/spermine N1-acetyltransferase (SSAT), a regulatory enzyme in a metabolic pathway that produces intracellular ROS [144] (Figure 3). The process is androgen-dependent, and JUND was also shown to be crucial for SSAT’s expression since JUND’s knockdown of LNCaP cells is unable to synthesize SSAT [144]. Hence, these studies describe a mechanism underlying ROS induction in androgen-dependent cells, able both to halt cell cycle progression and promote differentiation but also able to further promote carcinogenesis through the production of vast amounts of ROS via the polyamine oxidation pathway. A 2014 study further elaborated on the AR–JUND interaction and concluded that its inhibition by pharmaceuticals like GWARJD10 can reverse the expression of SSAT and thus limit the carcinogenic impact of aberrant ROS production, thus elucidating a novel tumor preventive target [145] (Figure 3).

Further studies have also credited JUND with pro-tumorigenic activity, mostly in androgen-dependent cells. A 2003 study by Zerbini et al. demonstrated that in androgen-independent PCa cells, IL6 is overexpressed, contributing to disease progression via its pleiotropic effects [135] (Figure 3). The transcriptional regulation of Interleukin 6 (IL6) was found to involve the constitutive activation of JUND, FOS-related antigen 1 (FOSL1; also commonly known as FRA1), Nuclear factor kappa B subunit 1 (NFKB1; also commonly known as NF-κΒ p50 subunit), and RELA proto-oncogene (RELA; also commonly known as NF-κΒ p65 subunit), thus crediting JUND with a pro-tumorigenic role [135]. In LNCaP cells, JUND was credited with a pro-proliferation role following treatment of the cells with hydrogen peroxide [136]. Hydrogen peroxide was reported to induce the activity of Pleiotrophin (PTN; also commonly known as Heparin Affin Regulatory Peptide, HARP), thus promoting the activation of FRA1, p-c-JUN, and JUND [136] (Figure 3). Stress-induced growth mediated by JUND was also reported in a 2009 study [137]. The authors reported how AP-1 is responsible for the promotion of growth and radioresistance in PCa, since following silencing of JUND and FRA1/2, proliferation and radioresistance ceased [137]. All three TFs were recognized as downstream targets of epidermal growth factor receptor (EGFR) and PI3K signaling, since the inhibition or loss of key pathway members leads to decreased AP-1 activity and reduced proliferation potential [137]. Pro-invasive activity mediated by JUND was also reported by Yamamoto et al. in a 2010 study [138]. The authors investigated how Wnt family member 5A (WNT5A) overexpression promotes invasion and demonstrated that it can phosphorylate JNK via PDK activation, ultimately leading to increased invasion and cell migration [138]. Following WNT5A activation, JUND was found to be recruited to the *matrix metallopeptidase 1* (*MMP1*) promoter, thus acting as a transcriptional regulator of the process [138] (Figure 3). A later study by Zerbini et al. in 2011 further fortified *JUND*’s role as an oncogene in PCa by showing that its inhibition leads to cell death [139]. The resulting apoptosis was shown to be growth arrest and DNA damage inducible alpha (GADD45A-) and growth arrest and DNA damage inducible gamma (GADD45G-) dependent, and silencing of JUND was also demonstrated to inhibit p38 and JNK MAPKs pro-survival signaling [139] (Figure 3). The study emphasized how JUND, as well as GADD45A and GADD45G, could be exploited as therapeutic targets, thus underscoring the significance of the described mechanism [139,146,147]. A 2016 study by Millena, T Vo, and Khan reported that JUND plays a crucial role in TGF-β-mediated growth regulation [24]. TGF-β was reported to downregulate JUND and cell proliferation of RWPE-1 and DU-145; however, this effect was not observed in PC-3 cells [24]. Following treatment with TGF-β, the forced overexpression of JUND led to increased proliferation and its knockdown led to a decline in proliferation rate [24]. Surprisingly, the authors report that knockdown of c-JUN or JUNB (under the influence of TGF-β) did not affect cell proliferation, while their overexpression led to a slower mitotic rate in DU-145 [24]. A 2019 study by Elliot et al. further supported JUND’s role in the proliferation of PCa cells by identifying gene subsets regulated by JUND that affect cell proliferation [140]. Knocking down JUND led to the downregulation of cell cycle regulators Peroxiredoxin 3 (*PRDX3*), Phosphoprotein enriched in astrocytes 15 (*PEA15*), Kinesin family member 2C (*KIF2C*), Cyclin dependent kinase 2 (*CDK2*), and the oncogene *MYC* while concomitantly promoting the expression of the cell cycle arrest promoter p21^Waf1/Cip1^ [24,140] (Figure 3). On the other hand, forced overexpression of JUND had opposing results, thus highlighting the protein’s role in PCa proliferation [140]. Since TGF-β has been found to act as both a tumorigenic factor and a tumor suppressor depending on the cellular context and the disease stage, it could have increased prognostic or even therapeutic value [148]. Another oncogenic role for JUND was recognized in AR-independent cancers in a recent study by Luo et al. [141]. The authors report that the silencing of Menin (MEN1) in PCa cells leads to nuclear translocation of JUND and Catenin beta 1 (CTNNB1; also commonly annotated as β-Catenin), which in turn promotes MYC expression and ultimately migration [141]. The cooperation of JUND with β-catenin was found to be crucial in the expression of EMT markers such as Cadherin 1 (CDH1 or E-cadherin), B lymphoma Mo-MLV insertion region 1 homolog (BMI1), Twist family bHLH transcription factor 1 (TWIST1), and Hypoxia inducible factor 1 subunit alpha (HIF1A) [141].

JUND has also been found to participate in transcription regulation loops with noncoding RNA molecules. A bioinformatics study conducted in 2020 by Xu et al. discovered that JUND and an upstream long-noncoding RNA positive regulator, LINC01600, are upregulated in radioresistant PCa tumors [142] (Figure 3). LINC01600 downregulation in PCa cell lines was shown to reduce JUND levels, further strengthening the suggested mechanism and crediting JUND with a role in radioresistance [142]. A more recent study associated JUND’s expression (as well as those of Forkhead box protein A1 [FOXA1] and serum response factor [SRF]) with the regulation of lncRNAs of high prognostic value, thus emphasizing its role in PCa progression [143]. Relations between JUND and micro-RNAs or long noncoding RNAs in the context of cancer are numerous [128,130,149,150,151], thus implying that further research is warranted in the context of urological malignancies.

## 4. Bladder Cancer

### 4.1. JUNB in Bladder Cancer

**Figure 4:**
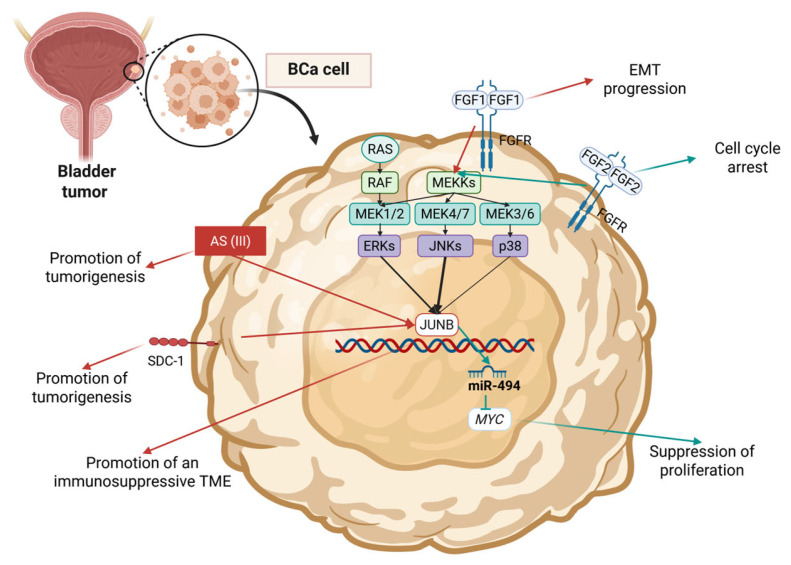
Key mechanisms of JUNB’s action and regulation reported in BCa studies. Blue arrows correspond to tumor-suppressing mechanisms, and red arrows correspond to oncogenic mechanisms. Created in BioRender. Kalampounias, G. (2025) https://BioRender.com/2aywg6m.

In bladder cancer (BCa), JUNB has been mentioned several times, mostly as a TF with tumor-promoting activity. In a 2010 study, JUNB was reported as a downstream target of Syndecan-1 (SDC1; commonly known as CD138), which has been repeatedly correlated to pro-tumorigenic cellular functions [152] (Table 3, Figure 4). JUNB was found to be downregulated following CD138 silencing in urothelial carcinoma cell lines, an outcome that impaired survival and led to induction of apoptosis [152]. A 2003 study reported that exposure to trivalent arsenic compounds (ASIII), such as methylarsine oxide (MAsIIIO) or iododimethylarsine (DMAsIIII), can induce the phosphorylation of cJun, JUNB, JUND, and FRA1 [153] (Figure 4). Mechanistically, the study reports that trivalent arsenicals promote the activation of ERK1/2 but not JNKs or p38 MAPKs, thus suggesting that the activation of JUNB is ERK1/2-dependent [153]. A 2008 study by Billottet et al. reported that JUNB plays a significant role in the EMT progression of BCa cells [154]. Specifically, Fibroblast growth factor 1 (FGF1) was found to act in multi-wave mode of transcriptional activation, and JUNB-mediated activation of transcription was found to be among the immediate targets [154] (Figure 4). A later study, by de Luna Vitorino et al., focused on Fibroblast growth factor 2 (FGF2), which has been credited with conflicting roles regarding the promotion of proliferation (Figure 4). In UM-UC-3 human urothelial carcinoma cells, FGF2 was found to exhibit anti-proliferative activity by affecting the intracellular transcriptional activity via the modulation of JUNB and FosB proto-oncogene (FOSB) [155]. The authors also confirmed that JUNB knockdown is not adequate to reverse the inhibition caused by FGF, which primarily affects S-phase progression [155]. A recent study by Chen et al.in 2022 credited JUNB with an important role in tumor microenvironment (TME) dynamics [156]. The study focused on immunosuppressive cell interactions between BCa cells and cancer-associated fibroblasts (CAFs), tumor-associated macrophages (TAMs), tumor-associated neutrophils (TANs), and regulatory T cells (Tregs) [156]. The expression of JUNB was found to be negatively correlated with those of chemokines, Major histocompatibility complex molecules (MHC), immunomodulators, immune checkpoint inhibitors, and other immunosuppressive factors, thus contributing to the formation of non-inflamed TME [156]. Given the fact that recent studies have credited JUNB with crucial roles in the formation of the TME and the regulation of immune responses [35,63,157,158], better understanding of the phenomenon could significantly increase the effectiveness of targeted therapies.

Finally, JUNB has also been credited with anti-metastatic effects. A 2019 study investigated the effects of a C8 fluoride derivative of cheliensisin A, namely ChlA-F, which was found to suppress BCa cells by promoting the expression of miR-494 [159]. The suggested underlying mechanism includes the stabilization of the JUNB mRNA, mediated by ELAV-like RNA-binding protein 1 (ELAVL1; also commonly known as HuR) [160], which in turn leads to increased transcription activity on the miR-949 promoter, due to JUNB binding [159]. The overexpressed miR-494 decreases the stability of MYC mRNAs and thus impairs its pro-metastatic activity [159].

### 4.2. JUND in Bladder Cancer

**Figure 5:**
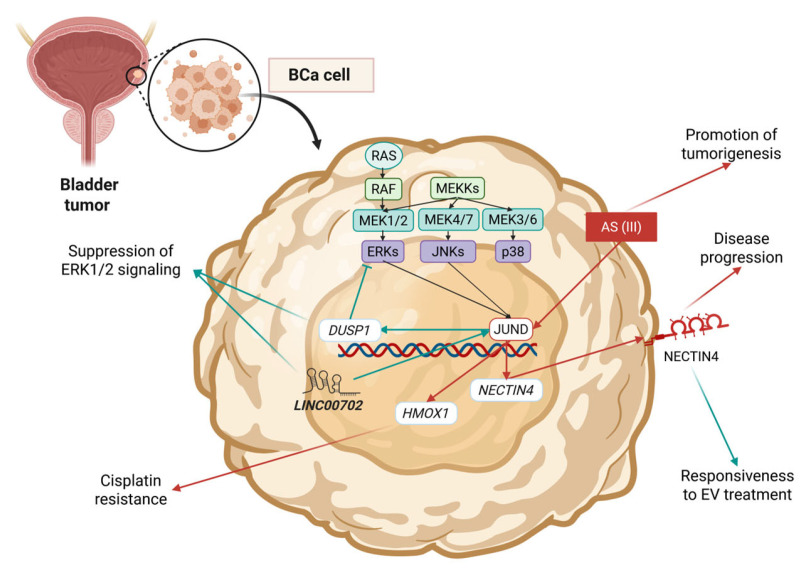
Key mechanisms of JUND’s action and regulation reported in BCa studies. Blue arrows correspond to tumor-suppressing mechanisms, and red arrows correspond to oncogenic mechanisms. Created in BioRender. Kalampounias, G. (2025) https://BioRender.com/dh92184.

Compared to JUNB, JUND has been reported less times in the context of BCa research, and only recently has it gained more attention. A 2020 study by Peng et al. demonstrated that in invasive BCa cells, JUND significantly contributes to cisplatin resistance by promoting antioxidant defense signaling [161]. More specifically, JUND was found to induce Heme Oxygenase 1 (HMOX1) expression and thus reduce the cytotoxic effects of cisplatin-provoked oxidative stress [161] (Table 4, Figure 5). A recent study by Xing et al. (2025) correlated the response of BCa to enfortumab vedotin (EV) therapy with diabetes [162]. Increased glucose levels were associated with lactate overproduction, which in turn was found to promote the lactylation of YTH domain containing 1 (YTHDC1) and its subsequent Ring finger protein 183 (RNF183)-mediated ubiquitination [162]. The decrease in YTHDC1 levels was found to impair JUND stability, thus affecting the expression of Nectin cell adhesion molecule 4 (NECTIN4), which is the target of EV therapy [162]. NECTIN4, an adhesion molecule highly expressed in several cancer types (including BCa), is a significant part of EV therapies [163,164]. Therefore, although JUND promotes its expression and thus tumor progression, increased JUND expression levels also determine responsiveness to EV [162] (Figure 5).

Finally, a 2022 study by Pan et al. focused on the role of lncRNA LINC00702 in BCa progression as a negative regulator and came up with an anti-tumor role for JUND [165]. LINC00702 was found to promote the expression of DUSP1 by recruiting JUND, and this mechanism was found to reduce proliferative potential by downregulating ERK1/2-mediated signaling [165] (Figure 5). On the contrary, an increase in the secretion of inflammatory factors by M2-TAMs was observed, thus also crediting JUND with TME-modulating activity [165]. Another study, by Li et al. in 2023, investigated the role of di-n-butyl phthalate (DBP) in BCa tumorigenesis and demonstrated that it promotes cancer progression by downregulating JUND, Dual specificity protein phosphatase 3 (DUSP3), and ATPase H+ transporting V1 subunit C2 (ATP6V1C2), while upregulating FOSB and Ras homolog family member Q (RHOQ) [166]. Therefore, the study underscored the role of *JUND* as a tumor suppressor gene opposing that of *FOSB* and *RAS* family proteins.

## 5. Renal Cancer

### 5.1. JUNB in Renal Cancers

JUNB has been reported several times in renal cell carcinoma (RCC). In 1992, Koo et al. studied the roles of c-JUN and JUNB in RCC and reported that c-JUN and JUNB have opposing activities in RCC (Table 5) [167]. The authors reported that transforming growth factor beta 1 (TGFB1) and Tumor necrosis factor (TNFA) inhibited the growth of RCC cells while also affecting the expression of c-JUN and JUNB [167]. Both TGFB1 and TNFA increased JUNB levels; however, only TNFA significantly increased c-JUN’s expression. Additionally, they reported that Interleukin 2 (IL2), Interleukin 4 (IL4), and Interferon gamma (IFNG) do not affect c-JUN/JUNB expression [167]. The study concluded that differential expression patterns of c-JUN/JUNB could modulate the anti-proliferative effects of TGFB1, TNFA, and IFNG [167].

**Figure 6:**
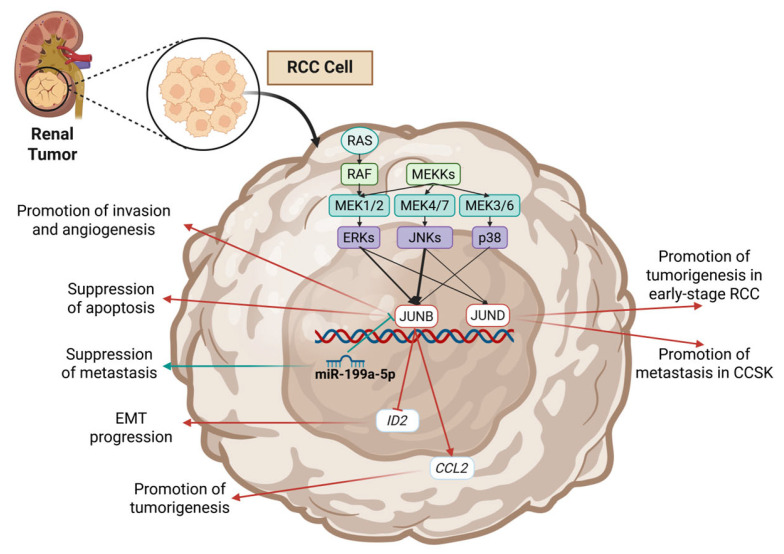
Key mechanisms of JUNB’s and JUND’s action and regulation reported in RCC studies. Blue arrows correspond to tumor-suppressing mechanisms, and red arrows correspond to oncogenic mechanisms. Created in BioRender. Kalampounias, G. (2025) https://BioRender.com/r9vqygj.

Later studies in renal cancers correlated JUNB with mostly oncogenic phenotypes. A 1997 study identified JUNB and JUND (as well as other AP-1 members like FRA1/2 and c-JUN) as TFs which are found overexpressed in very early stages of renal carcinogenesis [168]. A 2015 study by He et al. investigated dysregulated microRNAs in clear cell renal cell carcinoma (ccRCC) and identified several deviations from non-tumorous tissues [169]. JUNB and transforming growth factor beta receptor type 1 (TGFBR1) were identified as direct targets of a downregulated miRNA, miR-199a-5p, the overexpression of which effectively suppresses both JUNB and TGFBR1 mRNAs by interacting with their 3′ UTR regions [169] (Figure 6). In 2012, Gervasi et al. studied JUNB as a downstream target of TGF-β signaling and revealed that it is implicated in several pro-EMT cellular functions [170]. JUNB was found to suppress the expression of Inhibitor of differentiation 2 (IDF2) and contribute to tissue fibrosis by mediating the TGFβ induction of Fibronectin 1 (FN1), Fibulin 2 (FBLN2), Tropomyosin 2 (TPM2), and Integrin subunit beta 3 (ITGB3) [170] (Figure 6). A downstream effector of JUNB, the chemokine (C-C motif) ligand-2 (CCL2), was investigated by Arakaki et al. [171] in 2016 as a potential pharmaceutical target. CCL2 was found to be upregulated in ccRCC specimens, and its expression was correlated with the disease stage, the patients’ overall survival, and the macrophage infiltration [171] (Figure 6). In vitro modulations of CCL2’s expression were shown to affect cell proliferation and angiogenic potential, thus implying that JUNB and downstream molecules could be key targets of RCC treatments [171]. Upon comparison to the 1992 study by Koo et al. [167], it becomes evident that the role of JUNB can greatly shift depending on the subcellular context. TGF-b1 and TNFA are two of these examples, the administration of which elevated JUNB but did not cause any transformative results [167]. Moreover, the 1992 study did not investigate the expression of CCL2 in the cell lines, since this was shown to be the key effector mediating JUNB’s oncogenic activity according to Arakaki et al. [171].

**Table 5:** Roles of JUNB in renal and testicular cancers.

Cancer	Model	Role	Mechanism	Effect	Ref.
RCC	Patient-derived tissues and human RCC cell lines (R4, R6, and R11)	Tumor suppressor	JUNB exhibits opposing effects compared to c-JUN, and TGF-b1 increased the expression of JUNB	Inhibits tumorigenesis	[167]
RCC	Rat-derived tumors and rat RCC cell lines (LK9d, ERC-18, ERC27, ERC-31)	Oncogene	JUNB is overexpressed in early-stage rat RCC tumors compared to normal kidney tissue	Disease development	[168]
ccRCC	Patient-derived tissues and human ccRCC (786-O) and 293T cells	Oncogene	JUNB was downregulated by miR-199a-5p	Metastasis promotion	[169]
Kidney cancer	Mouse kidney epithelial cell line (MCT)	Oncogene	JUNB suppresses ID2	EMT promotion	[170]
ccRCC	Patient-derived tissues and human RCC cell lines (R4, R6, and R11)	Oncogene	JUNB exhibits oncogenic activity via one of its downstream effectors, CCL2	Promotes proliferation	[171]
RCC	Human RCC cell lines (786-O and A498)	Oncogene	c-RET and VHL mutations lead to increased JunB	Apoptosis suppression	[172]
RCC	Patient-derived tissues and human VHL-deficient RCC cell lines (786-O and A498)	Oncogene	JUNB promotes cell invasion and angiogenesis, while its suppression reverses the effect	Invasion promotion	[173]
Leydig cells	The mouse Leydig-cell tumor cell line (MA-10)	Oncogene	JUNB exhibits increased activity following treatment with hCG	AP1 activation	[174]

Abbreviations: RCC = renal cell carcinoma; ccRCC = clear cell renal cell carcinoma; EMT = epithelial-to-mesenchymal transition; hCG = human chorionic gonadotropin.

A separate group of studies has investigated the roles and functions of JUNB in Von Hippel–Lindau syndrome, a genetic condition with a strong correlation to certain tumors, including pheochromocytoma and renal cell carcinoma. JUNB was found upregulated following loss of the tumor suppressor gene VHL in RCC [175]. Lee in 2005 reported that VHL loss decreases the efficiency of ubiquitin-mediated degradation of several factors, including JUNB and HIF [172,175]. This study credited JUNB with a more prominent role in carcinogenesis, since its overexpression (due to VHL loss or decreased functionality) directly antagonizes cJun and downregulates apoptosis [175], a function that has been supported by other studies as well [176,177,178,179,180]. The significance of JUNB in RCC emergence and its relation to VHL were further reinforced by a later study in which a VHL variant, VHLX214L, was found to be associated with a Type 2A VHL syndrome, a subtype of VHL that increases RCC development risk. VHLX214L was found to be able to downregulate HIFα; however, JUNB downregulation was significantly impaired, and this failed suppression was suggested as a potential factor in VHL pathogenesis. Further research on JUNB’s role conducted in 2012 by Kanno et al. reported that in VHL-defective ccRCC, JUNB knockdown suppresses the invasive and angiogenic abilities of the malignant cells [173].

### 5.2. JUND in Renal Cancer

Up to this day, limited data exists about JUND’s role in the pathogenesis of renal malignancies; hence, the pure oncogenic role proposed by all of them could be biased. A 2023 study focused on a rare pediatric renal tumor, clear cell sarcoma of the kidney (CCSK), showed that tumors with the BCOR internal tandem duplication (ITD), which is the most common driver mutation in CCSK, exhibit increased JUND expression (as well as increased Fibroblast growth factor 3 [FGF3], Vascular endothelial growth factor A [VEGFA], Secreted phosphoprotein 1 [SPP1], and Adrenomedullin [ADM]) [181] (Table 6). All genes were correlated with increased MAPK signaling activity and were also found to be overrepresented in metastatic disease, underscoring their importance in disease progression. This study, together with the aforementioned 1997 study by Urakami et al., are the sole studies conducted so far about JUND in renal cancers, thus underscoring the need for more research in that direction [168,181].

## 6. Testicular Cancer

Finally, there is also a limited number of studies mentioning JUNB in testicular cancer. A 1998 study by Suzuki et al. reported that human chorionic gonadotropin (hCG) causes a rapid and transient expression of JUNB and JUND among other AP-1 family members (JUN, FOS, and FOSB) in Leydig cells (MA-10) [174]. A later study by Delbès et al. in 2009 studied the effects of the coadministration of bleomycin, etoposide, and cisplatin (BEP), a regimen used for testicular cancer treatment, on the gene transcription of germ cells [182]. The study showed that the treatment decreases sperm count and activates oxidative stress response genes [182]. The mRNA levels of JUNB and JUN were also found to be elevated in germ cells, and the authors observed that the phenomenon is directly correlated to oxidative stress damage, further underscoring the role of JUNB in stress responses and pro-survival mechanisms since the damaged cells do not undergo apoptosis but finally differentiate to abnormal spermatocytes [182].

## 7. Conclusions

All these findings combined indicate that both JUNB and JUND are implicated in several cellular processes associated with cancer, either as suppressors of its progression or as promoters. Besides their structural homology, both TFs also share several upstream regulators and downstream targets; therefore, their final result depends on several parameters of the subcellular context. The most significant parameter to be considered during result interpretation is the dimeric nature of AP-1. Since JUN proteins, even after their stabilization, do not act as monomers, the result from their activation is greatly affected by the other monomer participating in the AP-1 complex. Several studies credit JUNB or JUND with contradictory roles, even in the same cancer model or disease subtype, and this is a direct result of their participating in AP-1 complexes with a pro-transcription or suppressive activity. This subcellular environment is highly dependent on several factors, including epigenetic silencing/upregulation of JUN, ATF, or FOS genes, mutations, and external stimuli (both persistent and transient). Besides the presence of other AP-1 complex TFs, an important role is also played by differential post-translational modifications. Such alterations can modulate, increase, or decrease the TFs’ ability to interact with certain motifs and can thus significantly impact the observed effect. Phosphorylation at different sites is the most common mechanism; however, JUNB and JUND can also be ubiquitinated, SUMOylated, NEDDylated, and acetylated. Therefore, the presence of such enzymes and modifications of specific residues should be considered, as it greatly affects AP-1-induced gene expression. Phosphorylation alone is the most major contributor in this aspect of JUNB’s/JUND’s activity. Different MAPKs have been shown to produce differential results regarding JUN member activation; therefore, it is important to monitor a broad spectrum of upstream molecules in order to understand how JUN proteins function. Given the fact that all these mechanisms interact, the results are perplexingly divided into oncogenic and tumor-suppressing effects.

Regarding the TFs’ tumor-suppressing capacity, the major mechanisms reported can be categorized as either direct or indirect actions. Direct effects of JUNB/D include the antagonization of c-JUN in the transcriptional regulation of other oncogenes, the upregulation of cell cycle regulators like p16 and p21, the synergy with PTEN, and the transcription of anti-metastatic genes like KAI1. Additionally, interactions with JPDs lead to the formation of heterodimeric suppressive AP-1 complexes that usually have tumor-suppressing activity. On the other hand, indirect actions include the modulation of intracellular ROS levels by regulating the transcription of antioxidant enzymes and participation in complex tumor-suppressive axes by regulating the expression of noncoding RNAs. Nonetheless, most studies in the context of urological malignancies credit both JUNB and JUND with a mostly tumorigenic role.

Regarding JUNB, in urological cancers it is highly implicated in metastasis and secondarily in proliferation, controlling the expression of genes involved in cell adhesion, migration, and invasion. In several cases, JUNB was reported to cooperate with other TFs in favor of oncogenic transcription. Besides known interactions with JUN, FOS, or ATF family members that form AP-1 complexes, JUNB has been found to cooperate with other TFs (such as STAT3), promoting the expression of EMT markers, oncogenes like c-MYC, and immunomodulators. JUNB’s role in immune TME (in the context of urological tumors) is also a major aspect of its activity since it has been found to regulate associations of the tumor cells with TAMs and other immune cells. Similarly, JUND has been credited primarily with oncogenic activity, mostly centered around pro-proliferation signaling and stress-response regulation. In PCa, JUND has been found to regulate ROS levels and promote ROS-induced cell proliferation pathways while also regulating the expression of several noncoding RNAs. In BCa, data is scarcer; nevertheless, most reports credit JUND with a significant role in carcinogenesis, chemoresistance, and chemosensitivity. Although data is still limited, JUND seems to be a crucial part of the cell’s response to stress stimuli, including most chemotherapies, ROS, and several carcinogens; therefore, its expression and activation should be further investigated to shed light on underlying mechanisms of drug resistance and carcinogenic processes.

Since both TFs have been credited with such crucial involvement in cancer development and progression, their role as therapeutic, prognostic, diagnostic, and preventive factors should be further investigated. Agents targeting the interactions of JUN family members with other TFs, like the JUND-AR-targeting GWARJD10, may provide new tools against JUNB/D-mediated tumor progression and become part of novel targeted therapies of high specificity. Additionally, integrating JUNB/D screening with non-invasive state-of-the-art technologies such as liquid biopsy could provide novel prognostic factors of high significance, which could aid physicians in disease management and improve patients’ quality of life. Overall, in the context of urological cancers, the JUN transcription factor family seems to play a pivotal role in all disease stages, thus highlighting the need for more studies to expand current understanding and therapeutic capabilities.

## Figures and Tables

**Table 2:** Roles of JUND in prostate cancer.

Cancer	Model	Role	Mechanism	Effect	Ref.
PCa	Androgen-independent human PCa cell line (DU-145)	Tumor suppressor	Activity opposing c-JUN. Downregulation of FGF1, PTPN5, ADAM19, SERPINE1, CXCR7, MMP9, PLAU, and PTHLH	Migration suppression	[99]
PCa	Human PCa cell lines (DU-145, PC-3, LNCaP) and mice xenografts	Tumor suppressor	The expression of JUND increases as a result of JDP2	Tumorigenesis inhibition	[96]
PCa	Human PCa cell lines (DU-145, LNCaP)	Tumor suppressor	G_1_ arrest and JUND upregulation 72 h after exposure to androgen	Cell cycle arrest	[133]
PCa	Androgen-dependent human PCa cell line (LNCaP)	Tumor suppressor	JUND has an essential role in the androgen-induced increase in ROS levels	Increase in ROS levels	[134]
PCa	Human PCa cell lines (DU-145, PC-3, LNCaP)	Oncogene	Nuclear factor kappa B p50/p65, FRA1, and JUND promote the overexpression of IL6	Disease progression and drug resistance	[135]
PCa	Androgen-dependent human PCa cell line (LNCaP)	Oncogene	JUND is activated by PTN following stimulation with H_2_O_2_	Proliferation promotion	[136]
PCa	Androgen-independent human PCa cell lines (DU-145, PC-3)	Oncogene	JUND is upregulated and mediates EGFR- and PI3K-dependent signaling	Proliferation promotion and radio-resistance	[137]
PCa	Human PCa cell lines (DU-145, PC-3, LNCaP)	Oncogene	JUND is activated by WNT5A and upregulates MMP1	Invasion promotion	[138]
PCa	Androgen-independent human PCa cell lines (DU-145, PC-3)	Oncogene	JUND suppresses GADD45A/G and mediates JNK- and p38-dependent signaling	Cell death suppression and cell growth	[139]
PCa	Normal prostate (RWPE-1) and human PCa cell lines (DU-145, PC-3)	Oncogene	Knockdown of JUND expression suppresses proliferation, while forced overexpression of JUND increases it	Proliferation promotion	[24]
PCa	Androgen-independent human PCa cell lines (DU-145, PC-3)	Oncogene	JUND overexpression induces the expression of proliferation-associated genes, including c-MYC	Proliferation promotion	[140]
PCa	Androgen-independent human PCa cell lines (DU-145, PC-3)	Oncogene	JUBD and CTNNB1 are necessary for sustained MYC expression triggered by *MEN1* inactivation	Proliferation promotion	[141]
PCa	Bioinformatics study and patient-derived tissues	Oncogene	The expression of JUND is positively regulated by LINC01600 and is upregulated in radioresistant PCa	Radio-resistance	[142]
PCa	Bioinformatics study	Oncogene	JUND was found to participate in the regulation of genome-instability-associated lncRNAs with prognostic value	Genome instability	[143]
PCa	Androgen-dependent human PCa cell line (LNCaP)	Oncogene	JUND can induce the expression of SSAT, which produces excessive ROS	Carcinogenesis promotion	[144]
PCa	Androgen-dependent human PCa cell line (LNCaP)	Oncogene	GWARJD10 inhibits the JUND-AR interaction and represses the ROS-induced disease progression	Disease progression	[145]
CRPC	Patient-derived tissues	Oncogene	JUND was found to be a part of a transcription factor coordinated group (TFCG) enriched in the most aggressive type of disease	Disease progression	[104]

Abbreviations: PCa = prostate cancer; ROS = reactive oxygen species; CRPC = castrate-resistant prostate cancer; TFCG = transcription factor coordinated group.

**Table 3:** Roles of JUNB in bladder cancer.

Cancer	Model	Role	Mechanism	Effect	Ref.
UC	Human UC cell lines (UM-UC-2 and UM-UC-3)	Oncogene	JUNB is a downstream target of SDC-1	Promotes tumorigenesis	[152]
UC	Human UC cell line (UROtsa)	Oncogene	c-JUN, JUNB, JUND, and FRA1 are activated upon treatment with trivalent arsenic compounds	Increased AP1 DNA binding	[153]
BCa	Bladder carcinoma cell line (NBT-II)	Oncogene	JUNB is stimulated by FGF	Promotes EMT	[154]
BCa	Bioinformatics study, patient-derived tissues, and human BCa cell line (T24)	Oncogene	JUNB promotes the formation of an immunosuppressive TME	TME remodeling	[156]
UC	Human UC cell line (UM-UC-3)	Tumor suppressor	JUNB was found to be implicated in FGF2-induced cytostatic signaling	Suppresses proliferation	[155]
BCa	BCa cell lines (UM-UC-3 and HT-1197)	Tumor suppressor	JUNB stabilization promotes the expression of miR-494, which decreases c-MYC expression	Suppresses proliferation	[159]

Abbreviations: UC = urothelial carcinoma; BCa = bladder cancer; FGF = Fibroblast growth factor; SDC-1 = Syndecan-1; TME = tumor microenvironment.

**Table 4:** Roles of JUND in bladder cancer.

Cancer	Model	Role	Mechanism	Effect	Ref.
UC	Human UC cell line (UROtsa)	Oncogene	JUND is activated upon treatment with trivalent arsenic compounds	Increased AP1 DNA binding	[153]
MIBC	Bioinformatics study and patient-derived tissues	Oncogene	JUND upregulates the antioxidant defense via HMOX1	Cisplatin resistance	[161]
BCa	Patient-derived tissues, mouse models, and BCa cell lines (HT1376 and RT112)	Oncogene	JUND controls the expression of NECTIN4. However, its high expression determines EV therapy sensitivity	Treatment response modulation	[162]
BCa	Bioinformatics study, patient-derived tissues, human BCa cell lines (HT-1197, HT-1376, T24, and UM-UC-3), and the normal human urothelial cell line (SV-HUC-1)	Tumor suppressor	JUND is recruited by LINC00702 and upregulates DUSP1, which represses ERK1/2	Proliferation inhibition and modulation of TMI	[165]
BCa	Human BCa cell lines (T24 and UM-UC-3)	Tumor suppressor	DBP upregulates FOSB and RHOQ while downregulating ATP6V1C2, DUSP3, ORAI3, PLA2G15, PRICKLE3, TUBA1A, and JUND	Proliferation inhibition	[166]

Abbreviations: BCa = bladder cancer; UC = urothelial carcinoma; MIBC = metastatic muscle-invasive bladder cancer; DBP = di-n-butyl phthalate; EV = enfortumab vedotin.

**Table 6:** Roles of JUND in renal and testicular cancers.

Cancer	Model	Role	Mechanism	Effect	Ref.
RCC	Rat-derived tumors and rat RCC cell lines (LK9d, ERC-18, ERC27, ERC-31)	Oncogene	JUND is overexpressed in early-stage rat RCC tumors compared to normal kidney tissue	Disease development	[168]
CCSK	Bioinformatics study, patient-derived tissues, and HEK-293 cells	Oncogene	JUND is overexpressed in metastatic CCSK	Metastasis progression	[181]
Leydig cells	The mouse Leydig-cell tumor cell line (MA-10)	Oncogene	JUND exhibits increased activity following treatment with hCG	Cell growth	[174]

Abbreviations: RCC = renal cell carcinoma; CCSK = clear cell sarcoma of the kidney; hCG = human chorionic gonadotropin.

## Data Availability

No new data were created or analyzed in this study.
